# Rapid Pasteurization of Apple Juice Using a New Ultrasonic Reactor

**DOI:** 10.3390/foods9060801

**Published:** 2020-06-18

**Authors:** Zahra Moaddabdoost Baboli, Leonard Williams, Guibing Chen

**Affiliations:** 1 Center for Excellence in Post-Harvest Technologies, North Carolina A&T State University, The North Carolina Research Campus, 500 Laureate Way, Kannapolis, NC 28081, USA; zahramoaddab@gmail.com (Z.M.B.); llw@ncat.edu (L.W.); 2 College of Science & Technology, North Carolina A&T State University, 1601 E Market Street, Greensboro, NC 27411, USA

**Keywords:** new ultrasonic reactor, apple juice, microbial survival, stability, antioxidant activity, phenolic content

## Abstract

A new ultrasonic reactor was used to rapidly inactivate *Escherichia coli* and *Staphylococcus aureus* in apple juice. It was found that high pulp content made ultrasound less lethal to *S. aureus*, while it had no significant effect on *E. coli*. When the pulp free apple juice was ultrasonically processed, the 5-log reduction time was 35 s for *E. coli* at 60 °C and 30 s for *S. aureus* at 62 °C. Ultrasound treatment had no significant effect on antioxidant activity determined by 2,2-diphenyl-1-picrylhydrazyl (DPPH) radical scavenging activity, but it significantly increased the total phenolic content. The treatment also resulted in more stable juice with higher uniformity. During 28 d of storage at 4 °C, the total plate count in apple juice ultrasonically treated at 60 °C for 35 s remained around 1.00 log CFU/mL, whereas it was nearly zero for a stronger ultrasound treatment at 62 °C for 30 s. These values were much lower than those in the untreated one, which increased from 3.65 log CFU/mL to 8.36 log CFU/mL during the storage. At the end of the storage, the control and thermally treated apple juice lost almost 70% of antioxidant activity, whereas the ultrasonically treated juice only lost 20–40%.

## 1. Introduction

Fruits and vegetables are an essential part of a diversified and nutritious diet. A diet consisting of more than 400 g of fruits and vegetables per day has preventive effects against chronic diseases such as heart disease, cancer, diabetes, and obesity [1]. Among various types of fruits, apple is rich in phytochemicals [2,3] and consumed worldwide. Apple juice is the second most consumed juice in the United States [4]. Apple and its juice have shown anticancer effects in several human studies due to their high contents of flavonoids and polyphenols [5].

Due to the several outbreaks of illnesses from 1990s, the US Food and Drug Administration (FDA) requires all fruit juices to pass a mandatory HACCP plan to assure at least a 5-log reduction of a pertinent microorganism. From 1922 to 2010, *Escherichia coli* O157:H7 was responsible for most (199 cases and 2 deaths) of the foodborne illness outbreaks associated with apple juice in the United States [6] and, therefore, is considered as a pertinent microorganism in this juice [7]. *Staphylococcus aureus* is another microorganism of concern for food safety in apple juice, which has been justified in a reported study [8].

Thermal processing is the most used treatment for pasteurizing juices, but it causes a detrimental effect on nutritional quality and sensory attributes [9,10]. For this reason, nonthermal technologies have been developed to minimize the quality losses. Among them, ultrasonic processing has been extensively studied. Ultrasonic inactivation of microorganisms is associated with a phenomenon called cavitation, which can perforate and break microbial cells [11]. Many studies demonstrated that sonication could cause an increase in antioxidant activity or total phenolic contents of fruit juices with no or very low detrimental effect on color and texture compared with heat treatment. These juices included strawberry juice [12], orange juice [13], apple juice [14], etc. However, when a typical existing ultrasonic reactor is used, a long pasteurization time is needed to achieve the required 5-log microbial reduction, while inactivating some bacteria with a high tolerance to ultrasound is still a challenge. Moreover, for some fruit juices such as carrot and orange juices, ultrasonic processing did not result in sufficient microbial inactivation, leading to a short shelf life [15]. There were even some cases that showed sonication only slightly inactivated microorganisms [16,17].

Recently, we [18] developed a new batch ultrasonic reactor, which significantly shortens the pasteurization time of liquid foods. Using this reactor to inactivate *Escherichia coli* at 60 °C and *Staphylococcus aureus* at 62 °C in peptone water, a 5-log microbial reduction was achieved in 0.38 min and 0.55 min, respectively. In the present study, this new ultrasonic reactor was used to inactivate those two microorganisms in apple juice, and the objective was to investigate how the treatment influences the microorganisms, and antioxidant activity and total phenolic content in the juice during processing and their changes during storage at 4 °C.

## 2. Materials and Methods 

### 2.1. Preparation of Juices

Red delicious apples were purchased from a local market located in Kannapolis, North Carolina, USA. Fruits with no visible damage were selected and stored in a refrigerator for less than 24 h prior to juicing. The fruit samples were rinsed and sanitized by soaking in 200 ppm chlorine solution for 1–2 min based on federal regulation 21 CFR Part 173 [19], and then rinsed thoroughly and towel-dried. A slow juicer (Omega Juicer, Model VSJ843QR, Harrisburg, PA, USA) was used for making juice. The juice samples were filtered with 40 µm Fisherbrand TM cell strainers (Thermo Fisher Scientific, Waltham, MA, USA) to remove large pulp particles. The pH of the obtained apple juice was 3.9.

### 2.2. Preparation of Microorganisms

Nonpathogenic *Escherichia coli* 25,922 was demonstrated to be a potential surrogate organism for *Escherichia coli* O157:H7 [20] and therefore, it was used as a target microorganism in the present study. Nonpathogenic *Escherichia coli* 25,922 and *Staphylococcus aureus* 83,254 purchased from American Type Culture Collection (Manassas, VA, USA) were kept at −80 °C in tryptic soy broth (TSB; BD Bioscience, Franklin Lakes, NJ, USA) with 50% glycerol (Sigma-Aldrich, St. Louis, MO, USA) as freezer stocks. For each bacterium, 10 µL of thawed stock culture was inoculated in 5 mL of TSB and grown at 37 °C for 24 h. The culture was then spread to tryptic soy agar (TSA; BD Bioscience, Franklin Lakes, NJ, USA) plates and incubated overnight at 37 °C. A loop of the obtained bacterial colony was transferred into 5 mL of TSB and incubated in a shaker incubator (I24, New Brunswick Scientific, Enfield, CT, USA) at 37 °C and 60 rpm for 18 h to reach a cell density of around 10^9^ CFU/mL. One milliliter of this cell suspension was added to 9 mL of juice to dilute the bacterial density to about 10^8^ CFU/mL. Two milliliters of the inoculated sample were ultrasonically treated at a chosen temperature or thermally treated. The cell densities in a sample before and after treatment were determined using the plate count method described in a previous study [21]. To be effective, dilution of each original sample with 0.1% peptone water was arranged so that between 30 and 300 colonies of the target bacterium were grown in one plate.

### 2.3. Microbial Survival in Apple Juice during Ultrasonic Processing

#### 2.3.1. Ultrasound Treatment and Heat Treatment

**Figure 1:**
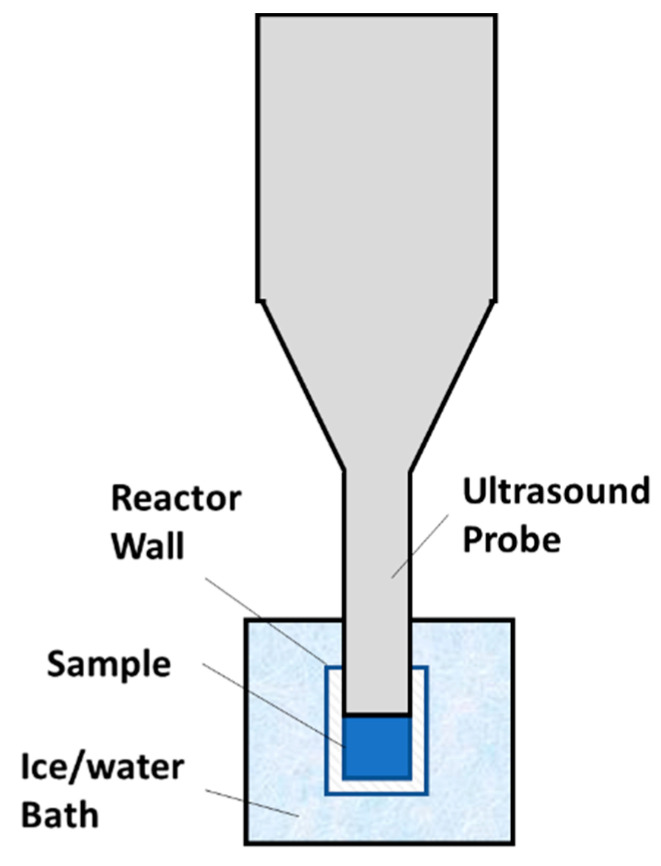
A cut view of the new ultrasonic reactor.

The ultrasound treatment and heat treatment were conducted following a previous study [18]. A cut view of the ultrasonic reactor was shown in Figure 1. A VCX 750 ultrasound generator (Sonics & Materials, Inc., Newtown, CT, USA) operated with a 1.3 cm probe was used to generate 20 kHz ultrasound. A Tygon E-3603 PVC tube (Saint-Gobain Performance Plastics, Akron, OH, USA) with an effective height of 1.5 cm and an interior diameter of 1.3 cm was used as a liquid processing vessel. The bottom end of the vessel was securely sealed using a rubber stopper. The ultrasound device was set at 45% amplitude for inactivation of *E. coli* and 50% amplitude for *S. aureus*. The generated average acoustic energy density (AED) was about 18.3 W/(mL liquid), which was estimated from the temperature increase during 5 s processing time following a reported method [22]. This AED value was about 3–9 times of those used in reported studies [23,24,25]. To maintain a sample’s temperature below a setpoint during processing, the vessel was inserted into an ice-water bath, which was frequently stirred to enhance heat transfer and ultrasound was applied in the continuous pulse mode, each pulse comprising of 5 s of processing time and 30 s of pause time, which were determined by preliminary tests. Temperature of a sample was measured at the beginning and end of each 5 s sonication treatment using a Digi-Sense Traceable^®^ Scientific Thermistor Thermometer with a micro probe (Cole-Parmer, Vernon Hills, IL, USA). The accuracy of the device is ±0.05 °C. A series of samples from the same batch of inoculated apple juice were processed for different number of pulses to obtain the microbial survival as a function of ultrasonic processing time.

Heat treatment at different constant temperatures was conducted to determine the highest temperature at which the sole heat treatment led to neglectable microbial inactivation during around 30 s. At each constant temperature, a series of sealed sterile glass capillary tubes (Wheaton, Millville, NJ, USA) filled with 250 μL of bacteria suspension were immersed in a precision shaking water bath (Thermofisher Scientific, Waltham, MA, USA). The come-up time of the measurement was around 30 s. At each chosen time point, two tubes were removed simultaneously from the water bath and immediately plunged into an ice-water bath to stop the inactivation.

#### 2.3.2. Effect of Pulp on Microbial Survival 

The prepared apple juice was centrifuged at 4000 rpm for 20 min using an Eppendorf 5810R centrifuge (Eppendorf AG, Hamburg, Germany) and the settlement was collected and dried in a freeze dryer (Labconco Corp., Kansas City, MO, USA). The obtained dry pulp was used to prepare apple juice with a high pulp level. The supernatant was treated as pulp free apple juice. The log reductions of *E. coli* and *S. aureus* in apple juice at three levels of pulp (1%, 0.4%, and 0%) were measured under processing conditions I and II listed in Table 1. Based on many measurements under the experimental conditions, the temperature at the end of each 5 s sonication was well controlled at around but no higher than its setpoint, and it was between 58.5 and 60 °C and 60.5 and 62 °C for conditions I and II, respectively. A typical temperature pattern for the continuous pulse sonication at setpoint 60 °C can be found in a previous study [18].

### 2.4. Thermal Processing

Apple juice was thermally processed at 72 °C for 16 s that is similar to the process typically carried out for milk pasteurization, which is adequate for pasteurizing apple juice at pH values of 4.0 or less [7]. Ten milliliter Pyrex screwed tubes filled with 4 mL of juice were immersed in a precision shaking water bath (Thermofisher Scientific, Waltham, MA, USA) at 72 °C for 16 s. The come-up time was measured to be 2 min and 15 s. Due to the contribution of the come-up time, the juice was over processed compared with the specified processing. The come-up time could be shortened by using metal tubes with a small diameter and shaking the tubes during processing. This thermal processing was listed as processing condition III in Table 1. Other time–temperature combinations are also used to process apple juice in the fruit juice industry [26,27].

### 2.5. Particle Size Analysis

Particle size distribution (PSD) in apple juice was analyzed using a blue wave laser particle analyzer (Microtrac, Montgomeryville, PA, USA). Prior to analysis, all samples were well shaken to ensure sample homogeneity.

### 2.6. Determination of Total Phenolic Content

The total phenolic content of apple juice was determined using the Folin–Ciocalteau (FC) reagent according to a reported method [28]. Briefly, 500 μL of apple juice sample was mixed with 7500 μL of methanol solution (methanol: water = 6:4). An aliquot (500 μL) of the obtained methanolic extract was then mixed with 2.5 mL of 10% (wt/wt) FC reagent in distilled water in a centrifuge tube. After 5 min, 2 mL of 7.5% (wt/wt) sodium carbonate aqueous solution was added. The mixture was then incubated at room temperature for 2 h, being vortexed several times during the incubation. Then, the mixture was centrifuged at 4000 rpm for 20 min. The supernatant was collected and absorbance at 760 nm was measured using a spectrophotometer (Model 2500, Shimadzu Corp., Kyoto, Japan). A standard curve was measured with serial gallic acid solutions. The total phenolic content was expressed as mg of gallic acid equivalent per liter of sample.

### 2.7. Measurement of Antioxidant Activity

The antioxidant activity of apple juice was measured by the DPPH radical scavenging activity using a reported method [29]. The stock solution was prepared by adding 2.5 mg of DPPH to 100 mL of methanol and kept at ambient temperature in a dark area. Then, 100 µL of the diluted juice sample with methanol solution (see Section 2.6) was mixed with 3.9 mL of DPPH solution. A control of DPPH solution was prepared using a combination of the DPPH solution and the same volume of methanol. The absorbance was determined at 515 nm using a spectrophotometer (Model 2500, Shimadzu Corp., Kyoto, Japan) after 2 h when the reaction reached a constant level. A calibration curve was prepared using a standard solution of Trolox. The antioxidant activity was expressed as mM of Trolox equivalent per milliliter of sample.

### 2.8. Measurement of Microbial Growth and Decrease in Antioxidant Activity and Total Phenolic Content during Storage

The control and treated apple juice samples were aseptically packaged into 50-mL screw-top polypropylene tubes and were analyzed on the 0th, 7th, 14th, 21st, and 28th day of storage at 4 °C. For microbiological analysis, the total plate count for mesophilic aerobic bacteria and fungi (molds and yeasts) in control and treated apple juice samples was determined by the pour plate method. To be effective, dilution of each original sample with 0.1% peptone water was arranged so that between 30 and 300 colonies of the target bacterium were grown in one plate. One milliliter of juice or related diluted solution was further pipetted into sterile Petri plates (dish) followed by addition of 15 mL of molten plate count agar (PCA; HiMedia, Mumbai, India) at 45 °C to each plate and mixed by “8” manner rotation of the plates. The PCA plates were incubated at 37 °C for 2 d. The total yeast and mold content were enumerated by additional incubation at room temperature for 3 d. The results were expressed as log colony-forming units (CFU) per milliliter of juice.

The DPPH radical scavenging activity and total phenolic content were determined using the above-described methods.

### 2.9. Statistical Analysis

All experiments were conducted in duplicate (*n* = 2) and each analysis was performed independently three times (a = 3). The data were analyzed by analysis of variance using the general linear models procedure (PROC GLM) in SAS^®^ 9.4 (SAS Institute, Cary, NC, USA) and expressed as the mean value ± standard deviation. The significant differences between mean values were determined by Tukey’s studentized range test at the 0.05 level.

## 3. Results and Discussion

### 3.1. Microbial Survival in Pulp Free Apple Juice during Ultrasonic Processing

**Figure 2:**
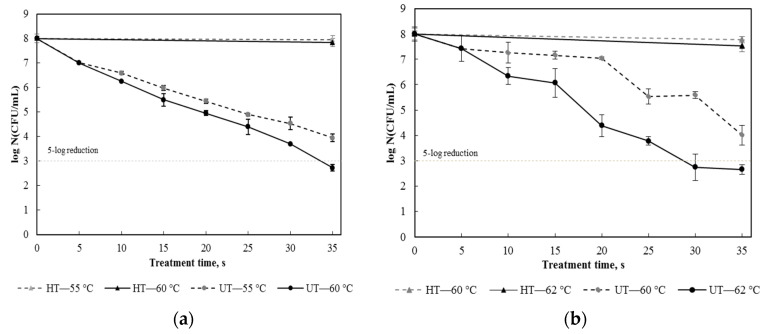
Survival curves of *Escherichia coli* (**a**) and *Staphylococcus aureus* (**b**) in apple juice after ultrasound treatment (UT) at different temperatures and heat treatment (HT) at the same temperatures.

Freshly squeezed pulp free apple juice inoculated with *E. coli* and *S. aureus*, respectively, was ultrasonically processed at different temperatures (55 and 60 °C for *E. coli*, and 60 and 62 °C for *S. aureus*) and thermally treated at the same temperatures. The resulting survival curves are shown in Figure 2. In screening tests, ultrasonic processing was performed at temperatures below 50 °C for *E. coli* and below 60 °C for *S. aureus.* At those temperatures, the inactivation effect of ultrasound was not enough to achieve a 5-log microbial reduction in one minute. Figure 2a shows that sonication at 60 °C quickly reduced the cell density of *E. coli* by 5 logs in 35 s while the nonthermal nature of the process was still maintained because the sole heat treatment at the temperature led to a microbial reduction less than 0.3 log; however, an ultrasound treatment at 55 °C for 35 s resulted in a 4-log reduction. The strengthening effect of temperature on ultrasonic inactivation of *E. coli* was also found in other studies [30,31]. Figure 2b shows that the survival of *S. aureus* under ultrasonic processing exhibited a similar trend, but a higher processing temperature was needed because *S. aureus* cells have a cocci shape and a Gram-positive cell wall structure, making them more resistant to the lethal effect of ultrasound [21,32]. Figure 2b also shows that the inactivation rate was very slow in the first 20 s of the ultrasound treatment at 60 °C. However, as the temperature increased to 62 °C, the inactivation rate significantly increased, and a 5-log reduction was achieved in 29 s. Based on these results, suitable ultrasonic processing conditions for *E. coli* and *S. aureus* were obtained and given in Table 1.

The results indicated that temperature and ultrasound had a synergic effect on the microbial inactivation rate. One reason was increasing temperature decreased the viscosity of the liquid medium and thus enhanced the generation of cavitation bubbles and strengthened violent bubble collapse [33]. The other reason was sublethal or higher temperatures could weaken the outer membrane of Gram-negative cells, making the cells less resistant to ultrasound [34]. The key role of temperature in ultrasonic processing has been investigated in many studies [35,36]. It is worth mentioning that when temperature reached a certain level, the involvement of ultrasound in thermal processing might not increase the microbial inactivation rate compared with the sole heat treatment [31].

### 3.2. Effect of Pulp on Microbial Survival during Ultrasonic Processing

Table 2 illustrates survival of *E. coli* and *S. aureus* in apple juice comprising of 0%, 0.4%, and 1% pulp during ultrasound treatment. Since typical commercial apple juice labeled as unfiltered contains around 0.4% pulp, these three levels of pulp represent pulp free, and low and high pulp contents, respectively. The particle size distribution in 0.4% and 1% apple juice was roughly similar. As shown in the table, pulp did not significantly influence survival of *E. coli*, whereas it was favorable for survival of *S. aureus*. This finding is only valid for the range of pulp contents examined. The upper limit of the pulp contents was much lower than that used in previous studies [37]. The reason was because the present experimental setup did not allow us to stir the juice during ultrasonic processing, leading to sedimentation of pulp at high pulp contents. A previous study demonstrated the lethal effect of ultrasound on total mesophilic aerobes in orange juice containing 1% and 10% pulp was not considerably different when ultrasonically processed [37]. For bacteria with smaller size such as *S. aureus*, the presence of pulp might protect the bacterial cells from ultrasonic wave and thus aid their survival [38].

### 3.3. Effect of Ultrasonic Processing on Pulp Particle Size

**Figure 3:**
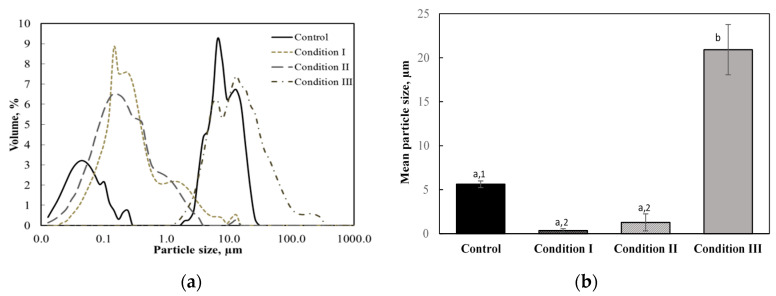
Particle size distribution (**a**) and volume mean diameters (**b**) of pulp particles in apple juice treated under different conditions. Values with different superscript letters are significantly different (*p* < 0.05). When the thermally treated juice (condition III) is not included in the statistical analysis, values with different superscript numbers are significantly different (*p* < 0.05).

The size distribution and volume mean diameters of pulp particles in apple juice processed under different conditions are presented in Figure 3. As shown in the figure, untreated apple juice exhibited a bimodal particle size distribution, one curve ranging from 0.013 to 0.243 µm with a peak at 0.05 µm, and the other ranging from 0.194 to 26.16 µm with a peak at 6.5 µm. The volume mean diameter of pulp particles in untreated apple juice was 5.6 µm. As shown in Figure 3a, the particle size distribution shifted toward smaller values with a peak at 0.14 µm and 0.17 µm for apple juice processed under condition I and condition II, respectively, whereas it shifted toward a larger value for the thermally processed juice under condition III. Accordingly, Figure 3b illustrates the volume mean diameters for juice processed under conditions I and II decreased, whereas that for the juice processed under condition III increased. This indicated ultrasonic waves broke down pulp particles, while heat treatment caused their aggregation. These results were in good agreement with previous findings [14,23,39].

Due to a significant reduction in the particle size, ultrasound treatment could result in more stable fruit juice with higher uniformity. Ertugay and Başlar [14] showed that apple juice with particle diameters less than 0.5 µm did not settle due to the Brownian motion and it was still stable with very little sediments after four months of storage. This was because high shear stress caused by ultrasonic cavitation could break colloidal pectin molecules in the juice [11,40].

### 3.4. Effect of Ultrasonic Processing on DPPH Value and Total Phenolic Content

Table 3 shows the effect of different treatment conditions on the DPPH value and total phenolic content. As shown in the table, overall, there was no statistical difference between samples treated under the three conditions except that sonication under condition II significantly increased the total phenolic content from 643.7 to 830.9 mg gallic acid equivalent/L. Previous studies showed inconsistent effects of ultrasonic processing on qualities of juices. Başlar and Ertugay [24] and Pokhrel et al. [41] found sonication did not change the examined properties of apple juice and carrot juice. However, other studies demonstrated the total phenolic content and antioxidant activity of apple juice were improved after ultrasound treatment [17,35,42], and the improvement was more remarkable at higher intensities or at longer treatment times [23]. The observed inconsistencies in these studies seemed related to the differences in the applied AED and temperature. When the AED is high enough, ultrasound treatment can release compounds from the cell walls and increase extractable compounds due to increased surface area of pulp particles. The AED applied in the present study was about 18.3 W/(mL liquid), which was much higher than those used in the reported studies [23,24]. Therefore, increased total phenolic content was observed. The extent of the increase had a strong positive relationship with temperature, indicating the synergistic effect between ultrasound and temperature. Table 3 also shows thermal processing at 72 °C for 16 s (treatment condition III) resulted in similar qualities of juice to those from mild ultrasonic processing under condition I. However, thermal processing at higher temperatures or for longer treatment times might cause more nutrient degradation [17,43,44].

### 3.5. Changes in Total Plate Count of Apple Juice during Storage

Table 4 depicts the total plate count of mesophilic aerobic bacteria, yeast, and mold in treated and untreated apple juice during 28 d of storage at 4 °C. As shown in the table, the microbial count in apple juice ultrasonically treated under condition I remained at around 1.00 log CFU/mL during the storage, whereas it was nearly zero or below the detection limit for a stronger ultrasound treatment under condition II and thermal processing under condition III. The total plate count in the treated apple juice was much lower than that in the untreated one, which increased from 3.65 log CFU/mL on the first day to 8.36 log CFU/mL on the 28th day of the storage.

In previous studies, the microbial count in ultrasonically treated fruit juices after a shorter time of storage at the same temperature was much higher compared with the present study. For example, the microbial count reached 6.5 log CFU/mL in ultrasonicated orange juice after 10 d of storage [45] and 3.1 log CFU/mL in carrot juice sonicated at 58 °C after 20 d of storage [25], which exceeded or was close to the limit of the satisfactory level (<4 log) of aerobic colony count for fruit juices [15]. Given longer storage times, the microbial count in these juices could easily pass the acceptance limit and cause food poisoning because the microorganisms grow rapidly once being recovered from injuries by the treatment. In some other studies, sonication did not significantly change the microbial population compared with the untreated one [16,17]. Table 4 shows there was 1.00 log CFU/mL population of microbes present in the apple juice subject to a mild ultrasound treatment under condition I. This number was far below the acceptance limit for mesophilic aerobic bacteria in fruit juices [46]. These microorganisms did not grow during the storage at 4 °C, indicating they were severely injured by the processing. Therefore, the new ultrasonic reactor was much more efficient in microbial inactivation than the traditional ones.

### 3.6. Changes in DPPH Value and Total Phenolic Content of Apple Juice during Storage

**Figure 4:**
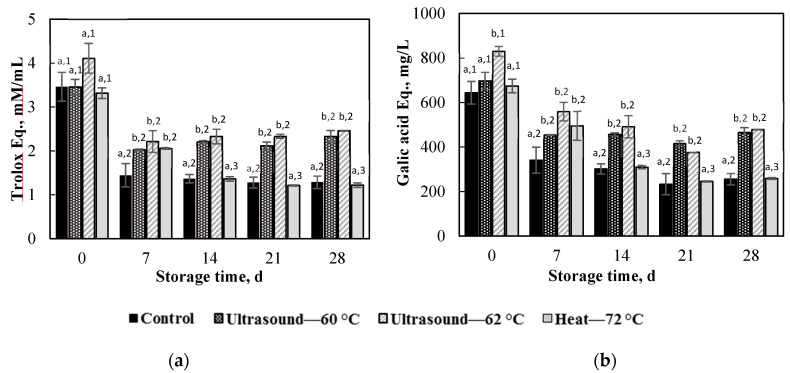
Changes in the DPPH radical scavenging activity (**a**) and total phenolic content (**b**) in apple juice treated under different conditions during storage at 4 °C. On the same day of storage, for juice treated under different conditions, values with different superscript letters are significantly different (*p* < 0.05). Under a treatment condition, for juice with different storage times, values with different superscript numbers are significantly different (*p* < 0.05).

Changes in antioxidant activity determined by the DPPH radical scavenging activity and total phenolic content of apple juice treated under different conditions during storage at 4 °C are depicted in Figure 4. The figure shows that values of DPPH and total phenolic content decreased with increasing storage time, but the degradation rates for ultrasonically treated apple juice were considerably lower than those of the control and thermally treated juice. Values of DPPH and total phenolic content dropped pronouncedly after one week of storage, while the degradation slowed down for longer storage times. After one week of storage, the DPPH values for apple juice ultrasonically treated under conditions I and II dropped 2 and 2.2 mM Trolox equivalent/mL, respectively, whereas the total phenolic contents decreased 455 and 560 mg gallic acid equivalent/L, respectively. However, values of DPPH and total phenolic content remained almost constant during the rest of the storage period. Similar observations were reported in previous studies [35,42]. On the other hand, values of DPPH and total phenolic content of the thermally treated juice kept decreasing in the second week of storage, but thereafter they remained almost constant. At the end of the storage, the control and thermally treated juice lost almost 70% of the DPPH radical scavenging activity, whereas the ultrasonically treated juice only lost 20–40%. The decreased degradation rates of bioactive compounds in ultrasonicated juice could be because ultrasonication effectively removes occluded oxygen from the juice, a critical factor influencing the stability of bioactive compounds [47].

## 4. Conclusions

This study clearly demonstrated that the new ultrasonic reactor [18] could be used to rapidly pasteurize apple juice in as short as 30 s under the experimental conditions examined. Temperature exhibited a strong positive effect on the microbial inactivation rate during ultrasonic processing. The pulp in apple juice did not affect the resistance of *E. coli* to ultrasound, but it substantially increased resistance of *S. aureus* to ultrasound. The ultrasonic processing of apple juice had no significant influence on antioxidant activity, but it significantly increased the total phenolic content, and stability and uniformity of the juice. During 28 d of storage at 4 °C, the total plate count in apple juice subject to a mild ultrasound treatment remained around 1.00 log CFU/mL, whereas it was nearly zero for a stronger treatment, indicating good microbiological stability of the processed juice. At the end of this storage, the control and thermally treated apple juice lost almost 70% of its antioxidant activity, whereas the ultrasonically treated one lost only 20–40%. For these reasons, the new ultrasonic reactor could be used to produce safe and nutritious apple juice and potentially other liquid foods with a reasonably long shelf life. It is noticeable that the volume of juice for each batch process is small for the current reactor. To address this issue, we are developing a laboratory-scale continuous flow ultrasonic reactor in which multiple ultrasonic probes with a tip diameter of 2 cm will be used. This system will have a volumetric flow rate of about 6 mL/s. The volumetric flow rate is directly proportional to the tip surface area of an individual probe and therefore, can be increased by increasing the probe tip diameter.

## Figures and Tables

**Table 1:** Ultrasonic and thermal processing conditions.

Processing Condition	Bacterium	Treatment	Temperature (°C)	Treatment Time (s)	Ultrasound Amplitude (%)
I	*E. coli*	Ultrasound	60	35	45
II	*S. aureus*	Ultrasound	62	30	50
III	-	Heat	72	16	-

**Table 2:** The effect of pulp on survival of *E. coli* and *S. aureus* in apple juice treated by ultrasound.

Bacterium	Treatment Condition	Pulp Content (%)		log(N_0_/N) (log CFU/mL)
*E. coli*		High	1	5.27 ± 0.14 ^a^
	I	Low	0.4	5.28 ± 0.13 ^a^
		Pulp free	0	5.04 ± 0.17 ^a^
*S. aureus*		High	1	4.30 ± 0.03 ^b^
	II	Low	0.4	5.26 ± 0.52 ^a^
		Pulp free	0	5.51 ± 0.26 ^a^

Note: Values with different superscript letters are significantly different (*p* < 0.05).

**Table 3:** The effect of different treatment conditions on the DPPH value and total phenolic content of apple juice.

Processing Condition	DPPH value (Trolox Equivalent, mM/mL)	Total Phenolic Content (Gallic Acid Equivalent, mg/L)
Control	3.46 ± 0.33 ^a^	643.7 ± 50.8 ^a^
I	3.45 ± 0.17 ^a^	696.9 ± 39.3 ^a^
II	4.11 ± 0.34 ^a^	830.9 ± 21.8 ^b^
III	3.31 ± 0.12 ^a^	674.2 ± 30.0 ^a^

Note: In the same column, values with different superscript letters are significantly different (*p* < 0.05).

**Table 4:** Microbial growth in apple juice treated under different conditions during storage at 4 °C.

Storage Time (d)	logN (log CFU/mL)			
	Condition I	Condition II	Condition III	Control
0	0.96 ± 0.17 ^a,1^	0.00 ± 0.00 ^b,1^	0.00 ± 0.00 ^b,1^	3.65 ± 0.40 ^c,1^
7	1.00 ± 0.02 ^a,1^	0.00 ± 0.00 ^b,1^	0.00 ± 0.00 ^b,1^	3.88 ± 0.63 ^c,1^
14	1.16 ± 0.28 ^a,1^	0.30 ± 0.00 ^b,1^	0.00 ± 0.00 ^b,1^	5.27 ± 0.49 ^c,2^
21	0.93 ± 0.04 ^a,1^	0.00 ± 0.00 ^b,1^	0.24 ± 0.34 ^b,1^	5.20 ± 0.10 ^c,2^
28	1.11 ± 0.05 ^a,1^	0.15 ± 0.21 ^b,1^	0.00 ± 0.00 ^b,1^	8.36 ± 0.53 ^c,3^

Note: In the same row, values with different superscript letters are significantly different (*p* < 0.05). In the same column, values with different superscript numbers are significantly different (*p* < 0.05).
